# Increased incidence and diverse manifestations of multiple evanescent white dot syndrome during the COVID-19 pandemic

**DOI:** 10.1038/s41598-024-63255-w

**Published:** 2024-05-30

**Authors:** Yong Yeon Song, Jung Tae Kim, Young Suk Chang, Min-Woo Lee, Sung Chul Lee

**Affiliations:** 1 Onnuri Eye Hospital, #325 Baekje-daero, Jeonju, Republic of Korea; 2 Department of Ophthalmology, Konyang University College of Medicine, Daejeon, Republic of Korea

**Keywords:** MEWDS, COVID-19, Vaccination, Incidence, Immunology, Environmental sciences, Diseases, Medical research

## Abstract

The advent of coronavirus disease 2019 (COVID-19) pandemic has affected the incidence and course of various diseases and numerous studies have investigated ocular involvement associated with COVID-19 and corresponding vaccines. In this study, we compared the incidence of multiple evanescent white dot syndrome (MEWDS) before and during the COVID-19 pandemic at a single center in Korea and analyzed the demographic and clinical features of patients with MEWDS presenting during the COVID-19 pandemic. We categorized patients with MEWDS into two groups according to date of diagnosis. Pre-COVID19 group included patients diagnosed during the pre-pandemic period (between March 11, 2017, and March 10, 2020), whereas post-COVID19 group included patients diagnosed during the pandemic period (between March 11, 2020, and March 10, 2023). 6 and 12 patients were included in pre-COVID19 group and post-COVID19 group, respectively. Among all hospital visits during the pre-pandemic and pandemic periods, 0.011% and 0.030% were due to MEWDS, indicating a significant increase during the pandemic (p = 0.029, B = 2.756). The annual incidence of patients with MEWDS in 2017–2022 were 0.73, 0.75, 0.78, 1.32, 2.49, and 2.07 per 10,000 population, respectively, corresponding to a significant increase (p = 0.039, B = 1.316). Our results imply that the incidence and manifestation of MEWDS are likely to become more diverse in the COVID-19 pandemic era.

## Introduction

Initially described by Jampol et al.^1^ in 1984, multiple evanescent white dot syndrome (MEWDS) is characterized by the unilateral presence of multiple gray-white dots at the retinal pigment epithelium or outer retina and foveal granularity. This rare disease predominantly affects young women, with an estimated annual incidence of 0.22 per 100,000 people^2^. Although its precise pathogenesis remains unclear, post-viral autoimmune or auto-inflammatory causes have been hypothesized^3^.

In March 2020, the World Health Organization (WHO) declared that the outbreak of coronavirus disease 2019 (COVID-19), caused by severe acute respiratory syndrome coronavirus 2 (SARS-CoV-2), had reached pandemic status^4^. As of August 30, 2023, approximately 770 million people worldwide had developed COVID-19, including almost 34 million reported cases in South Korea^5^. To safeguard individuals and contribute to herd immunity, the United States Food and Drug Administration authorized the emergency use of COVID-19 vaccines in December 2020. In South Korea, 86% of the population has received at least the second dose of COVID-19 vaccine^6^. Six COVID-19 vaccines have been authorized in Korea, ChAdOx1 nCoV-19 (Covishield™, Oxford-AstraZeneca) was first authorized on February 10, 2021, followed by BNT162b2 (Comirnaty™, Pfizer-BioNTech; March 5, 2021), Ad26.COV2.S (Jcovden™, Janssen; April 7, 2021), mRNA-1273 (Spikevax™, Moderna; May 21, 2021), NVX-CoV2373 (Nuvaxoid™, Novavax; January 12, 2022), and GBP510 (SKYCovione™, SK Bioscience; June 29, 2022). As of September 25, 2022, 128,710,064 doses of COVID-19 vaccines were administered: BNT162b2 (62.9%), mRNA-1273 (19.5%), ChAdOx1 (15.8%), Ad26.COV2.S (1.2%), NVX-CoV2373 (0.6%) and GBP510 (< 0.1%)^7^. Subsequent global efforts have effectively reduced the morbidity and mortality associated with COVID-19, leading the WHO Director General to declare the end of the COVID-19 public health emergency of international concern on May 5, 2023.

Numerous studies have investigated ocular involvement associated with COVID-19 and corresponding vaccines^8–11^. Case reports have described the occurrence of MEWDS after episodes of COVID-19 and vaccination against the disease^12–15^. The COVID-19 pandemic has influenced the incidence and course of certain diseases^16,17^. The present study explored the demographic and clinical characteristics of patients who developed MEWDS during the COVID-19 pandemic.

## Materials and methods

This study was conducted in accordance with the Declaration of Helsinki. The study protocol was approved by the Institutional Review Board/Ethics Committee of Konyang University Hospital, Daejeon, Republic of Korea (No. 2023–09-015). The need for informed consent was waived by the Institutional Review Board/Ethics Committee of Konyang University Hospital due to the retrospective study design.

### Study population

In this observational, single-center (Konyang University Hospital Eye Center), retrospective study, we comprehensively reviewed the electronic medical records of patients diagnosed with MEWDS who visited our hospital as an outpatient clinic between March 11, 2017, and March 10, 2023. The records were independently reviewed by two retina specialists (LSC and SYY) to identify patients who fulfilled the classification criteria for MEWDS published by the Standardization of Uveitis Nomenclature (SUN) working group in 2021^18^. These criteria include multifocal chorioretinal gray-white spots with foveal granularity; characteristic findings on fluorescein angiography (FA) or optical coherence tomography (OCT) extending from the retinal pigment epithelium into and/or through the ellipsoid zone into the outer nuclear layer of the retina; and absent to mild anterior chamber and vitreous inflammation. Patients were categorized into two groups according to date of diagnosis. pre-COVID19 group included patients diagnosed in the pre-pandemic period (between March 11, 2017 and March 10, 2020, total 3 years), whereas post-COVID19 group included patients diagnosed during the pandemic (between March 11, 2020 and March 10, 2023, total 3 years). Since we divided the two groups based on the date of the WHO pandemic declaration, the standard of one year was defined as the period from March 11 of that year to March 10 of the following year. For example, the year of 2017 was set from March 11, 2017 to March 10, 2018 and the year of 2022 was set from March 11, 2022 to March 10, 2023. The annual incidence of MEWDS cases was calculated as the proportion of MEWDS cases among the total number of patients who visited our clinic during each period. Demographic variables were compared between the groups, and specific cases from post-COVID19 group were analyzed in detail.

### Data analyses

Statistical analysis was performed using PASW Statistics software (version 20; IBM Corp., Armonk, NY, USA). Data are expressed as means ± standard deviations. Categorical variables were compared between groups using Fisher’s exact test, whereas continuous variables were compared using the Mann–Whitney U test. Poisson regression was used to compare the proportion of patients diagnosed with MEWDS between the pre-pandemic and pandemic periods and their annual incidence rate. P-values < 0.05 were considered indicative of statistical significance.

## Results

### Demographics

During the study period, 8 and 20 patients were evaluated for inclusion in pre-COVID19 group and post-COVID19 group, respectively; among these patients, 6 and 14 were diagnosed with MEWDS, respectively. In total, 3 patients were misdiagnosed and 5 did not fulfill the diagnostic criteria for MEWDS. Table 1 presents the baseline characteristics of patients of both group. Two of the patients were included in both groups but are only presented in pre-COVID19 group in Table 1. The mean ages were 36.5 ± 13.4 years in pre-COVID19 group and 37.58 ± 19.1 years in post-COVID19 group (p = 0.964). The proportions of female patients were 100% in pre-COVID19 group and 64% in post-COVID19 group (p = 0.260). The best-corrected visual acuity (BCVA) at presentation were 0.2 ± 0.3 in pre-COVID19 group and 0.2 ± 0.2 in post-COVID19 group (p = 0.750), and the corresponding BCVA at last follow-up were 0.0 ± 0.1 and 0.0 ± 0.0 (p = 0.820); these findings did not significantly differ between the groups. There were no significant differences in the time to improvement between pre-COVID19 group and post-COVID19 group (36.2 ± 14.2 and 33.6 ± 15.5 days, respectively; p = 0.733). There were no complications in ﻿pre-COVID19 group, whereas two patients developed complications in post-COVID19 group (p = 0.529).

**Table 1:** Baseline characteristics of both group.

	Pre-COVID19 group (n = 6)	Post-COVID19 group (n = 12)	P-value
Age	36.50 ± 13.43 (20–58)	37.58 ± 19.06 (13–78)	0.964^†^
Sex (M : F)	0 : 6	5 : 9^‡^	0.260*
Laterality (R : L)	4 : 2	5 : 6	0.620*
SE	− 4.98 ± 4.0	− 3.53 ± 4.27	0.456^†^
BCVA at presentation (LogMAR)	0.18 ± 0.30	0.18 ± 0.24	0.750^†^
Time for resolution (Days)	36.17 ± 14.22	33.64 ± 15.49	0.733^†^
Follow-up (Months)	8.53 ± 3.73	5.20 ± 6.20	0.241^†^
BCVA at last follow-up (LogMAR)	0.03 ± 0.05	0.02 ± 0.04	0.820^†^
Complication	0	2 (16.7%)	0.529*

*SE* Spherical equivalent, *BCVA* Best corrected visual acuity.

^†^Mann–Whitney.

*Fisher-exact test.

^‡^The recurrent cases were included.

### Incidence of MEWDS during pre-pandemic and pandemic period

**Figure 1:**
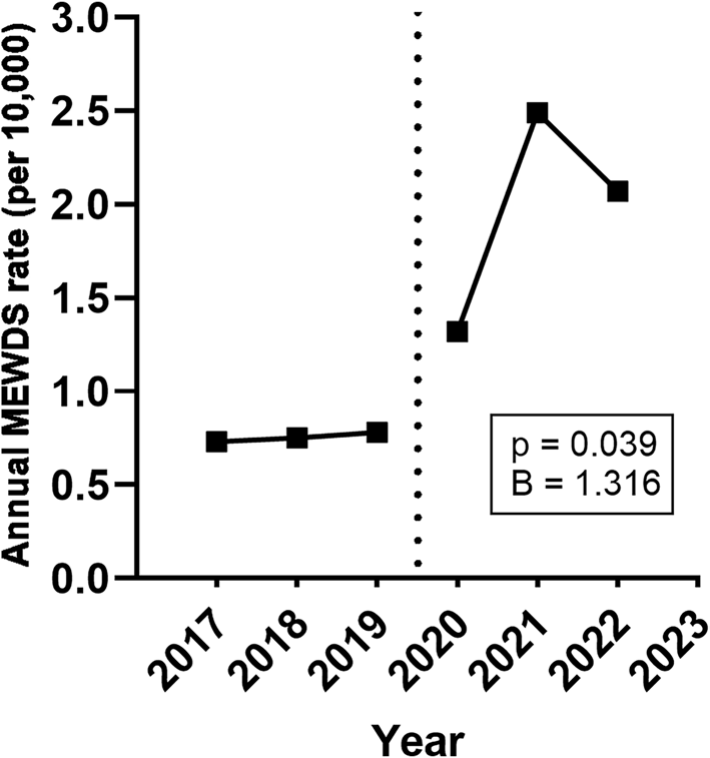
Annual incidence (per 10,000 people) of patients with multiple evanescent white dot syndrome (MEWDS) in our hospital. Each year represents the period from March 11 of the indicated year to March 10 of the next year. Dotted line indicates the declaration of the COVID-19 global pandemic. The annual incidence of MEWDS in 2017–2022 were 0.73, 0.75, 0.78, 1.32, 2.49, and 2.07 per 10,000 people, corresponding to a significant increase over the study period.

In the pre-pandemic period, 54,262 patients presented to the hospital; MEWDS was diagnosed in 0.011% of those patients. In comparison, 45,944 patients presented to the hospital during the pandemic; MEWDS was diagnosed in 0.030% of those patients, corresponding to a significant increase from the pre-pandemic period (p = 0.029, B = 2.756). The annual incidence of patients with MEWDS in 2017–2022 were 0.73, 0.75, 0.78, 1.32, 2.49, and 2.07 per 10,000 people, respectively, corresponding to a significant annual increase (p = 0.039, B = 1.316; Fig. 1).

### MEWDS cases in pandemic period

**Figure 2:**
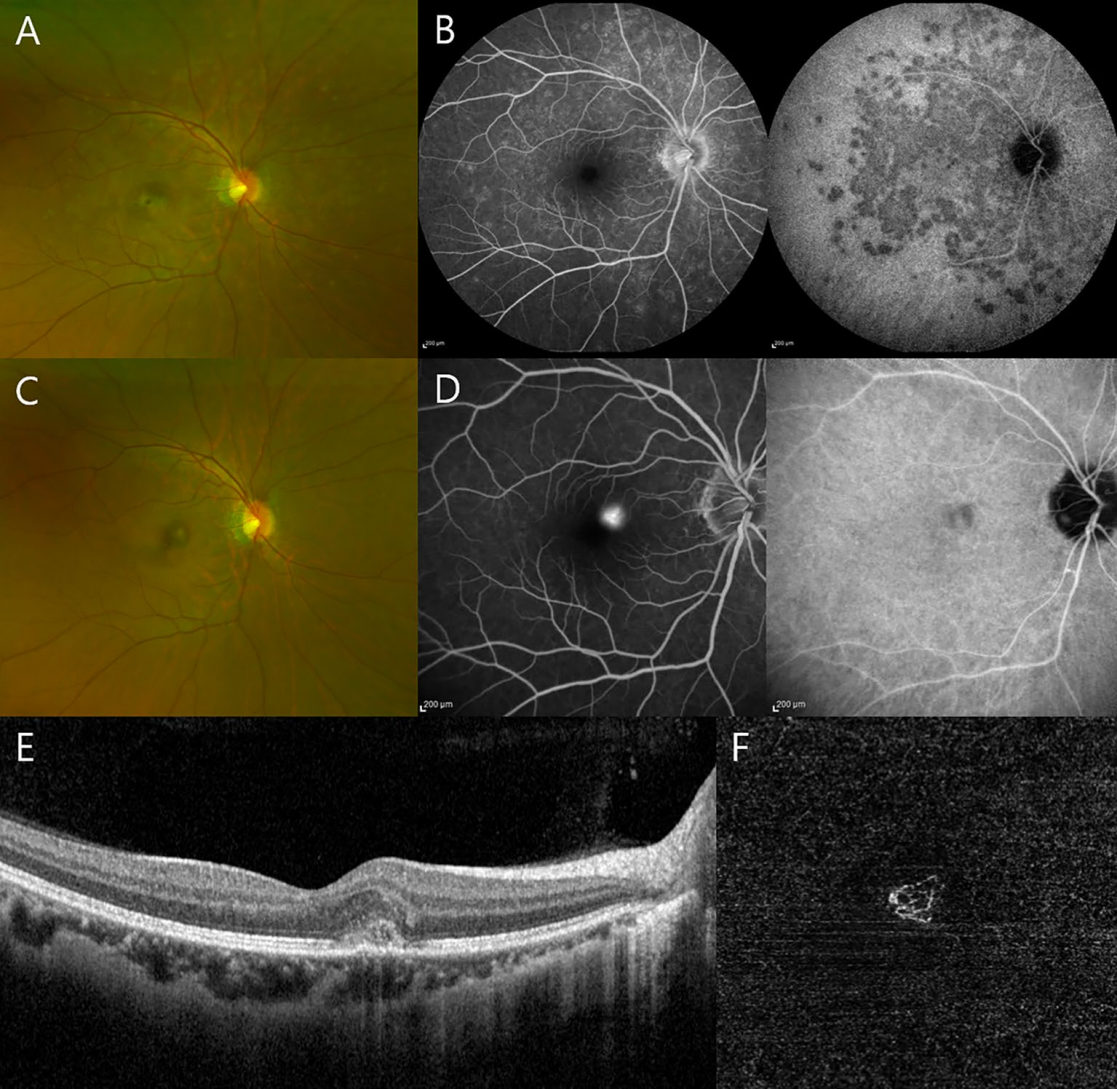
Case 2, a 40-year-old woman, developed macular neovascularization (MNV) 9 months after diagnosis with multiple evanescent white dot syndrome (MEWDS). (**A**) Fundus photography (FP) revealed multiple gray-white lesions at the posterior pole. (**B**) Fluorescein angiography (FA) demonstrated a “wreath-like” pattern of chorioretinal lesions, whereas indocyanine green angiography (IA) showed hypofluorescence of the dots during the late phase. (**C**) After 9 months, FP revealed resolution of the white dot-like lesions and development of a yellowish foveal lesion. (**D**) FA demonstrated a distinct hyperfluorescent spot and IA revealed hypofluorescence. (**E**) Optical coherence tomography (OCT) showed a subretinal hyperreflective material and a small amount of subretinal fluid. (**F**) OCT angiography demonstrated prominent MNV.

**Figure 3:**
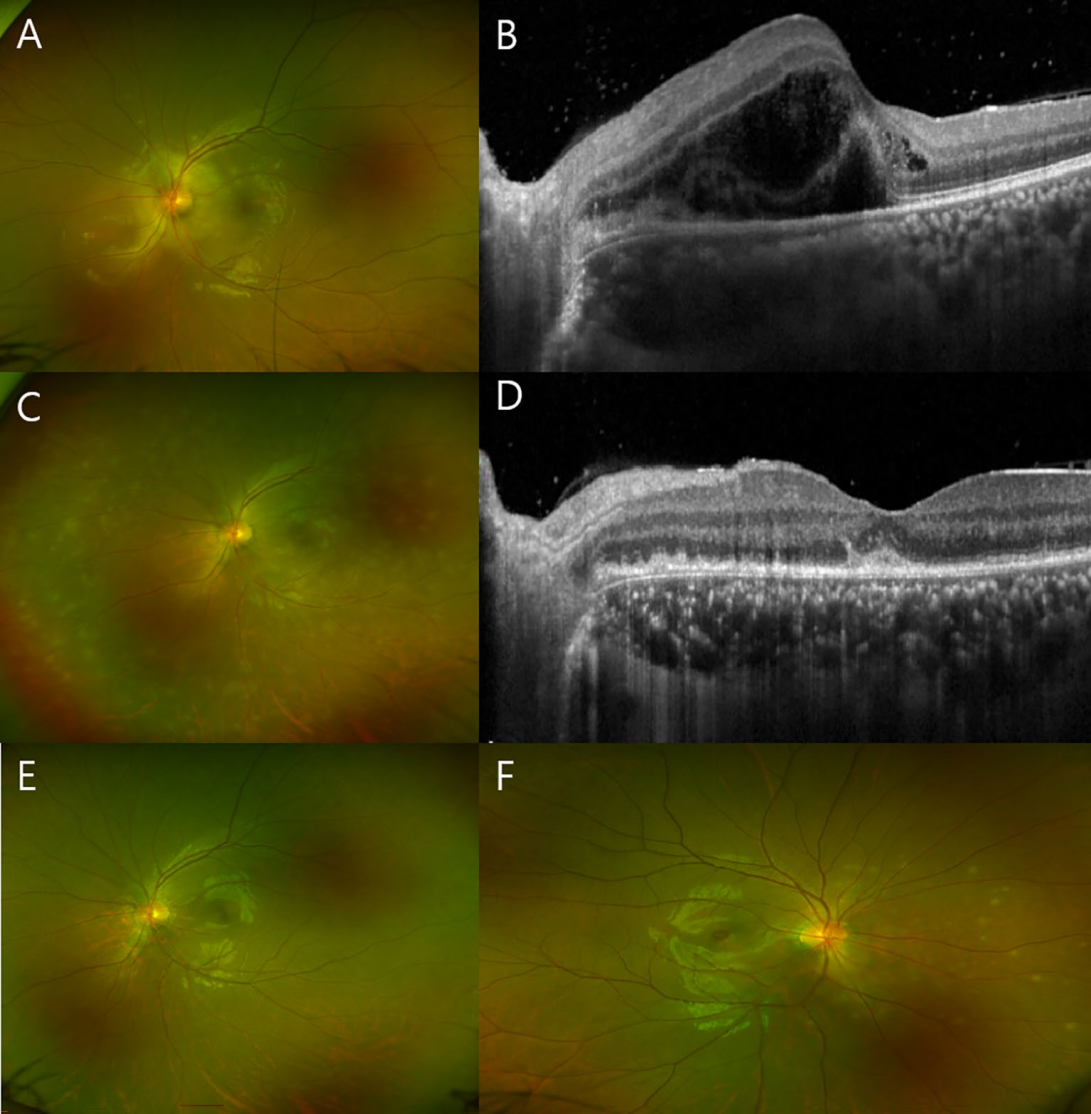
Case 9, a 23-year-old woman, developed bacillary layer detachement (BALAD). (**A**) At the first visit, fundus photography showed a whitish lesion in the juxtapapillary region. (**B**) Optical coherence tomography (OCT) demonstrated BALAD with subretinal fluid. (**C**) After 3 days, the lesion extended beyond the peripheral retina as white dot-like lesions, similar to multiple evanescent white dot syndrome (MEWDS). (**D**) OCT revealed disruption of the ellipsoid zone. (**E**,**F**) After 40 days, the lesion in the left eye disappeared; however, the patient experienced similar symptoms in the right eye, with white dot-like lesions on fundoscopy.

Table 2 presents the characteristics of patients with MEWDS during the pandemic period. Among the 14 patients, 2 developed MEWDS within 2 weeks after COVID-19, including the youngest patient in the group (Case 5; aged 13 years). Additionally, the oldest patient in the group (aged 78 years) developed MEWDS after COVID-19 vaccination. Two patients developed various features of MEWDS, including Case 2 who developed complicated macular neovascularization (MNV) after 9 months and successfully treated with anti-vascular endothelial growth factor (VEGF) (Fig. 2) and Case 9 who developed MEWDS in both eyes sequentially, rather than simultaneously, along with bacillary layer detachment (BALAD) with subretinal fluid (Fig. 3). As previously mentioned, two patients were included in both groups because of disease recurrence: Case 10 developed right eye MEWDS during the pre-pandemic period, followed by disease recurrence in the same eye 3 years later; Case 14 developed left eye MEWDS during the pre-pandemic period, followed by two disease recurrences in the same eye during the pandemic period.

**Table 2:** The summary of MEWDS cases in pandemic period.

Case no	Age	Sex (M: 0, F: 1)	Laterality (OD: 0, OS: 1, OU: 2)	Vaccine (Dose)	Time from Vaccine to Symptoms (days)	COVID-19 infection	Course	Initial BCVA	Final BCVA	Time for resolution (days)	Complication
1	35	1	0	−	−	−	Acute	0.0	0.0	50	–
2	40	1	0	−	−	−	Acute	0.0	0.0	18	MNV
3	20	0	1	−	−	−	Acute	0.5	0.1	21	–
4	31	1	1	−	−	−	Acute	0.0	0.0	60	–
5	13	0	0	−	−	+	Acute	0.4	0.0	55	–
6	53	1	1	M (1st)	−	−	Acute	0.0	0.0	30	–
7	78	0	1	P (2nd), P (B)	14	−	Acute	0.1	0.1	14	–
8	65	0	1	M (2nd)	−	−	Acute	0.5	0.0	28	–
9	23	1	2	P (2nd)	−	−	Acute	0.5	0.1	40	Bilateral(sequential). BALAD
10	33	1	0	AZ (2nd), P (B)	−	−	Recurrent	0.0	0.0	90	–
11	36	0	0	M (2nd)	−	+	Acute	0.0	0.0	24	–
12	32	1	0	AZ (2nd), P (B)	−	−	Acute	0.0	0.0	30	–
13	25	1	1	P (2nd)	−	−	Acute	0.1	0.0	35	–
14	20	1	1	−	−	−	Recurrent	0.3	0.2	28	Twice recurrence

*MEWDS* Multiple evanescent white dot syndrome, *M* Moderna, *P* Pfizer, *AZ* AstraZeneca, *B* 1st booster, *BCVA* Best corrected visual acuity, *MNV* Macular neovascularization, *BALAD* Bacillary layer detachment.

## Discussion

The COVID-19 pandemic has ushered in a “new normal,” significantly altering daily life and establishing new standards worldwide. The pandemic has also affected the incidence and course of certain diseases. Aydin et al.^16^ demonstrated increased frequency and altered clinical features of pediatric uveitis during the pandemic period, compared with the pre-pandemic period. Azar et al.^17^ demonstrated a significant increase in the incidence of acute macular neuroretinopathy from 0.66 per 100,000 people in 2019 to 8.97 per 100,000 people in 2020, which may be attributed to the COVID-19 pandemic. The present study was performed upon recognition of the increased number of hospital visits by patients with MEWDS during the pandemic period, compared with the pre-pandemic period. The annual visit rate of patients with MEWDS at our hospital also significantly increased from 2017 to 2022.

In COVID-19, SARS-CoV-2 targets endothelial cells through the angiotensin-converting enzyme 2 receptor, causing disruption of intercellular junctions, cell swelling, and impaired barrier function^19,20^. When these abnormalities occur in the peripapillary circulation of the retina, inflammation may involve the outer retinal layer. Ocular inflammation after COVID-19 vaccination is caused by molecular mimicry between uveal peptides and vaccine peptide fragments, antigen-specific cell and antibody-mediated hypersensitivity reactions, and adjuvant-induced inflammatory damage^10,21–23^. The pathogenesis of MEWDS, described as “common cold of the retina” by Tavallali and Yannuzzi^24^, involves the entry of viral agents into retinal photoreceptor cells located at the border of the optic nerve and ora serrata, triggering an autoimmune response^2,25^. Therefore, episodes of COVID-19 and vaccination against the disease can increase the risk of MEWDS. Bosello et al.^26^ demonstrated that the incidence of MEWDS was significantly higher in autumn, which may be due to the higher incidence of common influenza-like viral illnesses. We believe that increased viral transmission and vaccine use during the pandemic were associated with the increased frequency and altered clinical features of MEWDS.

### MEWDS following COVID-19

In post-COVID19 group, two patients developed MEWDS after COVID-19, including Cases 5 and Case 11. Case 5 was a 13-year-old boy who presented with a 5-day history of blurred vision in the right eye. Because he exhibited cough and chills for 10 days, he was tested by COVID-19 polymerase chain reaction; the result was positive. He had not undergone COVID-19 vaccination because of his young age. Case 11, a 36-year-old man, developed a scotoma 10 days after diagnosis with COVID-19. Despite receiving a second vaccination approximately 1 year prior, this was his first episode of COVID-19. Jain et al.^27^ reported the case of a 17-year-old boy who developed MEWDS after COVID-19; Zecevic et al.^12^ described MEWDS in a patient with concurrent COVID-19. Smeller et al.^13^ documented the case of a patient with COVID-19 who developed bilateral MEWDS.

### MEWDS following COVID-19 vaccination

Case 7, a 78-year-old man, was the oldest patient in post-COVID19 group; he developed blurred vision for 7 days. This patient had received the booster vaccination 2 weeks prior during admission to a nursing hospital. His clinical presentation was consistent with a clinical diagnosis of MEWDS and showed spontaneous improvement of the symptoms without any intervention after 2 weeks. Soifer et al.^14^ summarized 13 cases with COVID-19 vaccine-associated MEWDS and found that these patients had clinical features similar to the findings in patients who developed MEWDS after other vaccinations. Alhapshan and Scales reported the case of a 71-year-old female who developed MEWDS after COVID-19 booster vaccine administration which was similar with our case^28^.

All three cases of MEWDS associated with COVID-19 and vaccination against the disease occurred in male patients, resulting in post-COVID19 group included a higher proportion of male patients compared with pre-COVID19 group, although this was not statistically significant. Despite the small sample size in the present study, the proportion of female patients (64%) was lower than in previous reports (80–90%)^18,26,29^. Although the mechanisms that explain MEWDS occurring predominantly in women remain unclear, one hypothesis is that sex hormones cause women to have higher immunoglobulin levels and stronger immune responses than men, making them more prone to autoimmune or autoinflammatory reactions^30,31^. There are studies showing that COVID-19 and vaccination against the disease affect sex hormones^32,33^, and these changes may influence the incidence of MEWDS in male during the pandemic period. Furthermore, Cases 5 and 7 had outlier ages, which contributed to the wider age range in post-COVID19 group compared with pre-COVID19 group. Additional studies including larger number cases are needed to confirm hypothesis about this diversity.

Two patients with MEWDS developed complications during the pandemic period. Case 2 (Fig. 2), a 40-year-old woman, developed complicated MNV 9 months after diagnosis with MEWDS. OCT demonstrated exudates and OCT angiography revealed MNV. FA showed well-defined hyperfluorescent leakage. This exudative lesion was resolved after anti-VEGF injection and appeared quiescent at the final follow-up. MNV is a rare complication of MEWDS, and few cases have been reported. Parodi et al.^34^ performed a prospective study of four patients with MEWDS-related MNV, all of whom were successfully treated with anti-VEGF agents. Chen et al. ^35^ described four cases of MEWDS accompanied by type 2 MNV.

Case 9 (Fig. 3), a 23-year-old woman, complained of scotoma and blurred vision in the left eye, along with flu-like symptoms. Fundoscopy revealed juxtapapillary white lesions and OCT exhibited fluid accumulation as bacillary layer detachment (BALAD) with serous retinal datachment in corresponding lesion. Three days later, it was spreaded to the peripheral retina as white dot lesion in the manner characteristic of MEWDS. After 40 days, the left eye lesion had resolved but the patient developed similar symptoms in the right eye. Fundoscopy of the right eye revealed white dot-like lesions. Because the patient exhibited persistent fever and chills, she was evaluated for systemic diseases and diagnosed with Crohn’s disease. Fuganti et al.^36^ reported two cases of BALAD associated with acute central serous chorioretinopathy in patients after COVID-19. They described development of BALAD may be related to the systemic inflammatory condition observed in patients with COVID-19. In addition, several cases of Vogt–Koyanagi–Harada disease, in which BALAD is most frequently observed^37^, after COVID-19 and vaccination against the disease have been reported^38,39^.

These two complicated cases appear to be a complex product of various causes in the COVID-19 pandemic. Although the incidence of MNV in patients with MEWDS is unknown, this inflammation driven MNV is rare. Besides, this bilateral, sequentially developed MEWDS accompained with BALAD, was never reported before. These multifactorial factors, including infections, immune reactions and underlying diseases, may contribute to the development of this rare inflammatory complication. Especially for the Case 9, we speculated that the underlying Crohn’s disease and the COVID-19 pandemic led to a more severe disease course accompanying BALAD. However, in some ways, it is statistically predictable that as the incidene of MEWDS increases, the number of cases showing various clinical findings increases.

Two female patients developed disease recurrence and were included in both groups. Case 10 experienced MEWDS before the pandemic, followed by disease recurrence in the right eye during the pandemic. Case 14 experienced the first episode of MEWDS before the pandemic and two disease recurrences during the pandemic. Ramakrishnan et al.^29^ reported that 10 of the 73 patients with MEWDS (14%) in their study experienced disease recurrence; this proportion was similar to the present study, in which 2 of the 18 patients in both groups experienced recurrence (11%). Soifer et al.^14^ described a case of recurrent MEWDS after COVID-19 vaccination, and they summarized two similar cases that were previously reported. These findings suggest that repeated exposure to viruses and vaccines during the pandemic can predispose an individual to MEWDS recurrence.

It is challenging to prove a causal relationship between MEWDS and episodes of COVID-19 or vaccination against the disease. However, previous studies have demonstrated an increased risk of uveitis after COVID-19 vaccination. Testi et al.^10^ described 70 patients who exhibited ocular inflammation within 14 days of COVID-19 vaccination, among whom 12.9% had posterior uveitis. Rabinovitch et al.^15^ reported 21 cases with uveitis after COVID-19 vaccination: 19 with anterior uveitis and 2 with MEWDS. The investigators suggested that a causal relationship between these conditions was likely based on the short time interval (< 30 days) between vaccination and uveitis; however, according to the WHO classification of adverse drug reactions, all patients had probable or possible causality. In South Korea, more than 87% of individuals have received COVID-19 vaccines and numerous people have experienced COVID-19. Moreover, it is challenging to determine the number of asymptomatic COVID-19 cases. Our results suggest that the complex changes associated with the pandemic, including multiple infections and immunological responses, contributed to the increased incidence of MEWDS and associated variable clinical findings.

This study had several limitations. First, the sample size was small due to the low incidence of MEWDS. To overcome this limitation, we acquired data over a long period of time (i.e., 6 years). Second, this was a retrospective study. To minimize variability across data, we applied the recent SUN classification criteria for MEWDS. Third, some cases had missing information related to COVID-19 or vaccination against the disease, making it challenging to determine a causal relationship. Fourth, data were collected from a single center, limiting the generalizability of our results to other populations. Nevertheless, our study is clinically valuable because we demonstrated an increased incidence of MEWDS during the pandemic period, compared with the pre-pandemic period. Future multicenter studies with a larger sample size are needed to clearly characterize the relationship between COVID-19 and MEWDS.

In conclusion, we observed a significantly greater number of patients with MEWDS presenting to our hospital during the COVID-19 pandemic period, compared with the pre-pandemic period. Furthermore, the annual incidence of patients with MEWDS significantly increased over the 6-year study period. Considering the increased exposure to viruses and vaccines during the COVID-19 pandemic, the demographic and clinical features of MEWDS are likely to become more diverse.

## Data Availability

The datasets generated during and/or analysed during the current study are available from the corresponding author on reasonable request.
